# Case report: Dermatofibrosarcoma protuberans of the foot: What steps can we take?

**DOI:** 10.1016/j.ijscr.2023.108667

**Published:** 2023-09-09

**Authors:** Miguel Matias, Miguel Verissimo, Raquel Barbosa, Diogo Casal

**Affiliations:** Plastic Reconstructive Surgery Department, Centro Hospitalar Universitário Lisboa Central, Lisbon, Portugal

**Keywords:** Dermatofibrosarcoma, Plastic surgery, Foot Cancer, Case report

## Abstract

**Introduction and importance:**

Dermatofibrosarcoma protuberans (DFSP) is a rare soft tissue sarcoma affects mainly the trunk and proximal limbs. Clinically, it typically presents as an asymptomatic plaque or nodular-like lesion that progresses slowly before entering a rapid growth phase. DFSP exhibits a low potential for metastasis, mainly in cases where fibrosarcomatous transformation occurs, but it has a high rate of local recurrence. Diagnosis of DFSP is often delayed, and it is challenging to establish without performing a biopsy and histologic analysis. The mainstay treatment for DFSP is surgical wide excision with free margins, although this can be challenging depending on the location of the tumor.

**Case presentation:**

We report a rare presentantion of dermatofibrosarcoma protuberans according to the SCARE guidelines. The patients main concern was the slow evolving mass on the dorsum of the foot that at presentation was 1x1cm. The biopsy showed a dermatofibrosarcoma protuberans. A radical excision involving ray amputation of the 2nd and 3rd finger provided a 1 cm clear margin.

**Clínical discussion and conclusion:**

This case shows how an inconspicuous nodule in an uncommon area can be the primary manifestation of a serious condition.

## 1 Background

Dermatofibrosarcoma protuberans (DFSP) was first described by Sherwell and Taylor in 1890 [1,2]. It was first named as DFSP by Hoffman in 1925 [4,5] and it was recognised early on as a cutaneous tumor with tendency to develop protruding nodules and with high recurrence rates.

DFSP is a rare, locally aggressive cutaneous sarcoma, originating from dermal fibroblasts, with a high propensity for local recurrence and a low risk of metastasis. It tends to infiltrate deep locally, invading fascia, muscle, periosteum and bone [5]. Clinically, it typically presents as an asymptomatic plaque or nodular-like lesion that progresses slowly before entering a rapid growth phase. DFSP exhibits a low potential for metastasis, mainly in cases where fibrosarcomatous transformation occurs, but it has a high rate of local recurrence. Diagnosis of DFSP is often delayed, and it is challenging to establish without performing a biopsy and histologic analysis.

Due to its slow-growing and insidious nature, DFSP is often misdiagnosed clinically, resulting in a delayed diagnosis that allows disease progression [6].

In recent years, numerous advances have been made regarding its clinical and histological features. Consequently, new treatment approaches are being developed, including innovative surgical procedures and emerging targeted pharmacologic regimens [2].

In this article, we present a clinical case that showcases an atypical presentation of this condition in our academic enter, and we use it to review the current surgical treatment options and present the epidemiology, pathogenesis, clinical features, diagnosis, staging and prognosis, along with a case report.

## 2 Epidemiology

According to the latest epidemiological studies, the incidence of DFS is estimated to be between 0.8 and 5 cases per million people per year worldwide, accounting for less than 0.1 % of all malignant tumors and less than 1 % of all soft-tissue sarcomas [2,5].

DFSP of the foot and toes is even rarer, with only a few cases reported in the literature [[10], [11], [12], [13]].

It can occur in people of any age, being diagnosed from congenital cases to patients with more than 90 years old [2]. The age spectrum differs in several articles, but they all recognize that it most commonly affects young to middle-aged adults [2,3,5]. There is a low incidence of DFSP in children, but the tumor has been reported in infants as young as a few months old. DFSP in children is more commonly associated with the infantile subtype, which tends to be more aggressive and has a higher rate of recurrence compared to the adult-onset subtype. The slow growth of these tumors has led to a hypothesis that they may originate during childhood but may not become noticeable until early adulthood [16].

Female patients are slightly more affected than man according to some studies, although gender ratio is roughly one [7,8]. DFSP incidence is also higher in African-Americans than Caucasians, although this evidence is not in agreement between different studies [2,5,9].

In recent population-based studies, the five-year relative survival rates are high (98–100 %), not differing significantly by race or sex [8].

## 3 Pathogenesis

DFSP is a relatively rare tumor, and its exact cause is not well understood. The key tumor pathogenesis is the chromosomal translocation involving the COL1A1 and PDGFB genes, t(17; 22) (q22; q13), being present in over 90 % of cases. This results in the fusion of type I collagen, at 17q22, and platelet-derived growth factor beta, at 22q13, promoting the proliferation of fibroblasts and myofibroblasts by the overexpression of platelet-derived growth factor beta. This is believed to play a role in the development and progression of DFSP.

Histologically, several variants have been described, including the classic subtype, the pigmented (Bednar tumor), the myxoid, the granular cell, the atrophic DFSP, DFSP with fibrosarcomatous areas (DFSP-FS), DFSP with areas of giant cell fibroblastoma (GCF), DFSP/DFSP-FS with foci of myoid/myofibroblastic differentiation, and sclerosing/sclerotic DFSP [2]. The classic subtype is the most common and is characterized by a spindle cell proliferation with a honeycomb or storiform pattern. The pigmented variant is characterized by the presence of melanin pigment within the tumor cells, while the fibrosarcomatous variant is characterized by increased cellularity and mitotic activity, as well as the presence of a more disordered architecture.

Some data report that multiple factors are involved in the development of DFSP, including oncogenes, tumor suppressor genes and immunodeficiency, although further investigation is required to understand the relationship of these risk factors.

## 4 Clinical features

Dermatofibrosarcoma protuberans (DFSP) typically presents as a slow-growing, painless, firm, and often flat or slightly elevated skin lesion. The lesion may be pink, brown, or skin-colored, and it may have an irregular shape or surface. DFSP often has a characteristic “honeycomb” or “storiform” appearance under the microscope.

DFSP can occur in any part of the body but is encountered most commonly on the trunk (40–50 %), generally on the chest and shoulders, and extremities (30–40 %), mainly in the proximal portion. In 10–15 % of the cases, it affects the head and neck, generally the scalp, cheek, and supraclavicular area. When occurs on the scalp, it may be mistaken for a benign cyst or tumor. It has been reported that childhood DFSP has a greater tendency toward acral location.

The tumor may have satellite nodules in the surrounding skin, and it may extend deeply into the subcutaneous tissue [14].

DFSP rarely metastasizes, but it can invade surrounding tissue and recur locally after surgical excision. Local recurrence is common if the tumor is not completely excised with clear margins. In some cases, DFSP can transform into a more aggressive sarcoma known as a fibrosarcoma.

## 5 Diagnosis and staging

The diagnosis of dermatofibrosarcoma protuberans (DFSP) is usually made through a combination of clinical examination, imaging studies, and histopathological analysis.

Although a presumptive hypothesis can be made clinically, the definitive diagnosis is made by incisional or excisional, biopsy procedure.

### 5.1 Clinical examination

A Dermatologist or Plastic Surgeon will usually begin by performing a thorough physical examination of the skin lesion, noting its size, shape, color, texture, and any associated symptoms such as pain or tenderness. They will also examine the surrounding skin for signs of satellite nodules or other abnormalities.

### 5.2 Imaging studies

DFSP is identified radiologically by the presence of a soft tissue mass that involves the skin and subcutaneous adipose tissue, and it is characterized by the absence of mineralization [16].

Imaging studies such as ultrasound, magnetic resonance imaging (MRI), or computed tomography (CT) may be used to evaluate the depth and extent of the tumor and to identify any areas of invasion or recurrence.

For planned operative procedures for DFSP, MRI has emerged as the preferred modality for defining the margins due to its superior resolution compared to CT. Additionally, MRI is more useful than CT in imaging DFSP because it allows for the production of images with different imaging protocols such as T1 and T2. On T1-weighted images, DFSPs usually appear hypointense to fat, while on T2-weighted images, they appear hyperintense to isointense to fat [17,18].

### 5.3 Histopathological analysis

The definitive diagnosis of DFSP is made through a biopsy of the lesion and subsequent histopathological analysis of the tissue sample. A pathologist will examine the sample under a microscope to evaluate the appearance and pattern of the tumor cells, as well as the presence of any characteristic markers or genetic mutations.

Immunohistochemistry may be used to confirm the diagnosis and to distinguish DFSP from other benign and malignant skin lesions that may have similar clinical and histopathological features. In particular, the presence of the characteristic chromosomal translocation involving the COL1A1 and PDGFB genes is highly specific for DFSP.

Once a diagnosis of DFSP is confirmed, further imaging studies may be done to assess the extent of the tumor and to determine the best treatment approach. For the initial workup searching for distant metastases is usually unnecessary because they are exceedingly rare in DFSP, except for cases with suspected metastasis upon clinical examination or recurrent disease, and for DFSP with fibrosarcomatous transformation features [15].

There is no standard staging system for DFSP. In general and according to European Consensus-based guidelines, the primary tumor is considered stage I, lymph node metastasis is stage II and distant metastasis stage III [8].

Based on this classification, a recent study by Cleveland Clinic in 2020 proposed a modified stage system in order to enhance diagnosis, workup and treatment: Stage I: Non-protuberant lesions including atrophic or sclerotic plaque, macula or small nodules; Stage II: Protuberant primary tumor; Stage IIA: Superficial tumor: without invasion of the underlying fascia; Stage IIB: Deep tumor: either superficial to the fascia with infiltrating the fascia or occurred beneath the superficial fascia; Stage III: Lymph node metastasis; Stage IV: Distant metastasis to other organs [5].

## 6 Treatment and prognosis

The primary treatment for DFSP is surgical excision with clear margins, which means removing the entire tumor along with a margin of normal tissue around it to ensure that no cancer cells are left behind. The goal of surgery is to completely remove the tumor and prevent local recurrence.

In the case of invaded margins during initial surgery for DFSP, it is recommended to perform re-resections whenever possible until clear margins are achieved. Before definitive reconstruction, it is important to thoroughly assess all surgical margins. Surgery for DFSP should be carefully planned, taking into account the size of the tumor, the type of margin control required, the tumor's location, and cosmetic issues to determine the most appropriate surgical procedure [8].

There are various surgical techniques available for treating dermatofibrosarcoma protuberans (DFSP). These include wide local excision (WLE) with clear margins, Mohs micrographic surgery (MMS) and partial or total amputation in cases where the tumor is situated on the digits.

According to the latest European Consensus-based Guidelines, Mohs micrographic surgery (MMS) or related variants is the treatment of choice with evaluation of all peripheral and deep margins, particulary in recurrence-prone regions, as it can significantly lower the risk of recurrence of DFSP, compared with WLE. While MMS is preferred, it is not widely available, and in such cases, a larger lateral safety margin of 3 cm is recommended.

Recurrence rates following these procedures were compared in a comprehensive retrospective meta-analysis involving 684 DFSP patients from 2008 to 2018, which showed recurrence rates of 9.10 % and 2.72 % for WLE and MMS, respectively [8]. The Mayo Clinic experience illustrated a recurrence rate of 30.8 % following WLE and 3.0 % with MMS, with MMS being the preferred choice for relatively small DFSPs in cosmetically sensitive regions such as the face, scalp, and neck [20].

The width of tumor-free margins is crucial for complete excision for both procedures, but there is no agreement on the optimal width. According to the latest NCCN guidelines, lateral margins of 2–4 cm from the tumor and excision of the investing fascia to remove any infiltrating tumor are recommended for WLE. Recurrence rates with margin width varied widely, but 1.0 to 1.5 cm margins are safe for most DFSPs on the trunk or extremities, and MMS should be selected for cosmetically sensitive regions [19].

Dissection of the tumor bed should be based on the infiltrating depth of the tumor, with deep tumors requiring excision of the underlying investing fascia and superficial tumors requiring direct excision without dissecting underlying fascia. DFSP on digits can be challenging due to limited space and complex structures, and MMS may be preferable to WLE in these cases. Partial or total amputation may be necessary if the tumor extends to the periosteum, but if the patient is opposed to amputation, enucleation can be performed with further referral to oncologists for radiation therapy and/or targeted therapy.

Regardless of the technique used, immunohistochemical analysis is useful to evaluate the tumor margins of the excised material [8].

In cases where surgical excision is not possible or would result in significant functional or cosmetic impairment, other treatment options may be considered, including radiation therapy or targeted therapy.

### 6.1 Radiation therapy

Radiation therapy may be used to treat DFSP in cases where complete surgical excision is not possible or in cases where the tumor has invaded surrounding tissues. Radiation therapy uses high-energy beams to kill cancer cells and may be given before or after surgery. It is generally well-tolerated, but it may cause side effects such as skin irritation, fatigue, and nausea.

### 6.2 Targeted therapy

Targeted therapy is a newer approach to treating DFSP that involves using drugs that specifically target the genetic mutations that drive tumor growth. The most commonly used targeted therapy for DFSP is imatinib mesylate, which is a tyrosine kinase inhibitor that blocks the activity of the platelet-derived growth factor receptor (PDGFR) that is overexpressed in DFSP. Imatinib mesylate has been shown to be effective in shrinking or stabilizing DFSP tumors in many patients, although it is not curative and must be taken long-term.

In cases of recurrent or metastatic DFSP, treatment options may be more limited, and the goal of treatment is typically to control the tumor and relieve symptoms. In these cases, targeted therapy with imatinib mesylate or other drugs may be used, along with radiation therapy or surgery as appropriate.

### 6.3 Clinical case - identification

The following case is reported in accordance to the SCARE guidelines [21]. A 48-year-old woman, born in Brasil and residing in Portugal was referred to the Plastic Surgery Consult by dermatology due to a slow growing mass on her left foot that presented no symptoms. She had a history of epilepsy but didn't take any medication. She didn't smoke or drink. She has no family history of cancer. She presented a mass on the dorsum of the feet that didn't interfere with walking.

### 6.4 Clinical case – clinical findings and diagnostic assessment

The patient presented with a single mass on the fibular aspect of the dorsum of the 2nd finger of the right foot measuring 1x1cm. The mass was solid, rubber hard and was immobile in relation to the deeper tissue plains. There were no palpable lymphatic ganglions in the popliteal fossa or inguinal region. The differential diagnosis of a soft tissue tumor with malignant characteristic includes Sarcoma and Osteosarcoma.

An incisional Biopsy was performed that showed a DFSP.

The limb and cervico toraco Abdominal CT Scan showed no distant sites of disease.

A surgical excision was performed with a 4 cm margin. The definitive histopathology exam was delayed due to the lengthy process of decalcification.

**Fig. 1:**
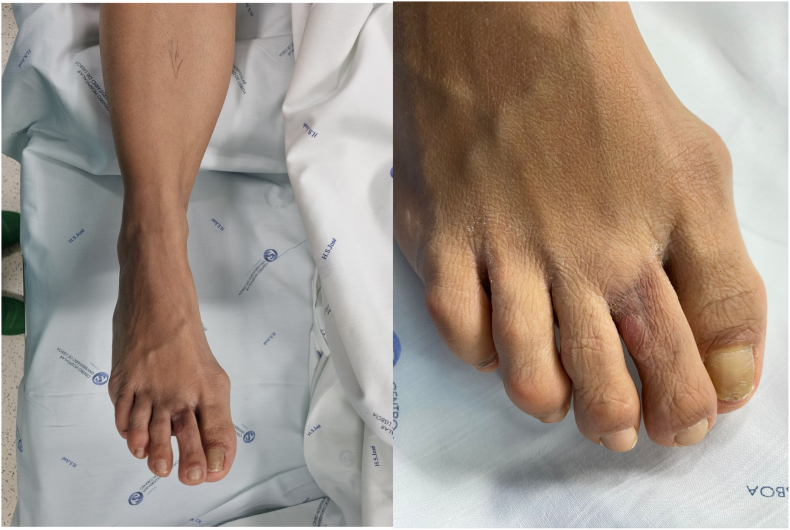
Pre operative lesion.

The timeline is showed in Fig. 1.

**Figure:**
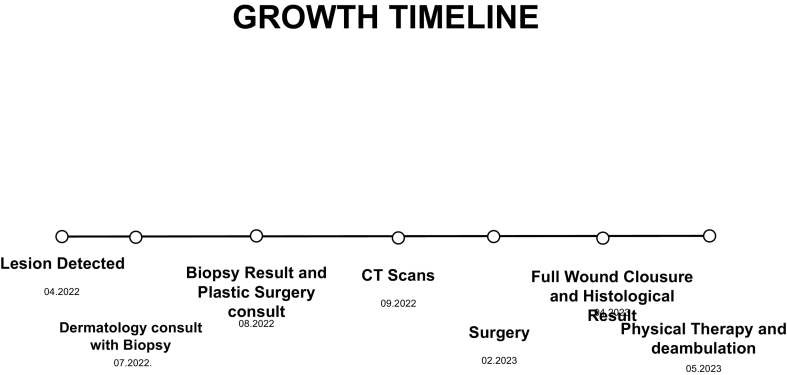

### 6.5 Clinical case – therapeutic intervention

The patient was proposed to surgery under general anesthesia. Due to the wide margins required, the proposed treatment was an harmonious ray amputation of the 2nd and 3rd finger of the right foot. The patient was operated in a single stage, in Dorsal decubitus with a Leg tourniquet at 350 mmHg. The surgical team was made of a Senior Plastic Surgery Assistant and two Residents. The oncological phase was started by the marking of a wedge incision made on the plantar and dorsal aspect of the foot, that encompassed the 4 cm margin. The first step was to identify the neurovascular pedicle of the fibular side of the 1st finger and the branches of the superficial peroneal nerve. The deep peroneal nerve crossed the margins of safety of the tumor. Dissection progressed from superficial to deeper planes until the second and third metatarsal. A Bone Saw was used to make a clear cut in the bone and the bone edges were trimmed to allow a rounder surface. In the reconstructive phase the deep intermetatarsal ligament was sutured by Nylon 3.0. The volar and dorsal flaps were closed as much as possible and in the remaining area a fine thickness skin graft was applied with a tie over bolster dressing. There were no intra operative findings that required a change in the operative plan.

In the post operative period the patient was kept in dorsal decubitus for 1 week, followed by a month of aided walking with crutches. Thromboprophylaxis with 40 mg enoxaparin was employed. The graft doner zone was opened at day 5 post op and kept with alternate day dressings above the primary greasy gauze until it was epithelized. The bolster dressing was opened at D15 and residual areas were closed by secondary intention.

In the post operative stage, dressing changes addressed residual areas of graft digestion. At D2 post op neuropathic pain developed in the deep peroneal nerve territory and was managed with incremental doses of gabapentin.

At two months post op the patient is able to walk and has started physiotherapy in order to adapt to the new plantar stepping surface.

**Fig. 2:**
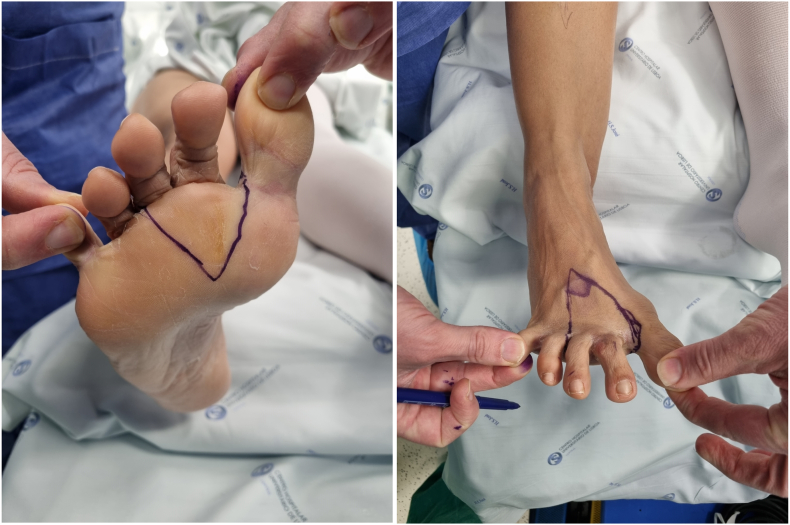
Pre surgical markings.

**Fig. 3:**
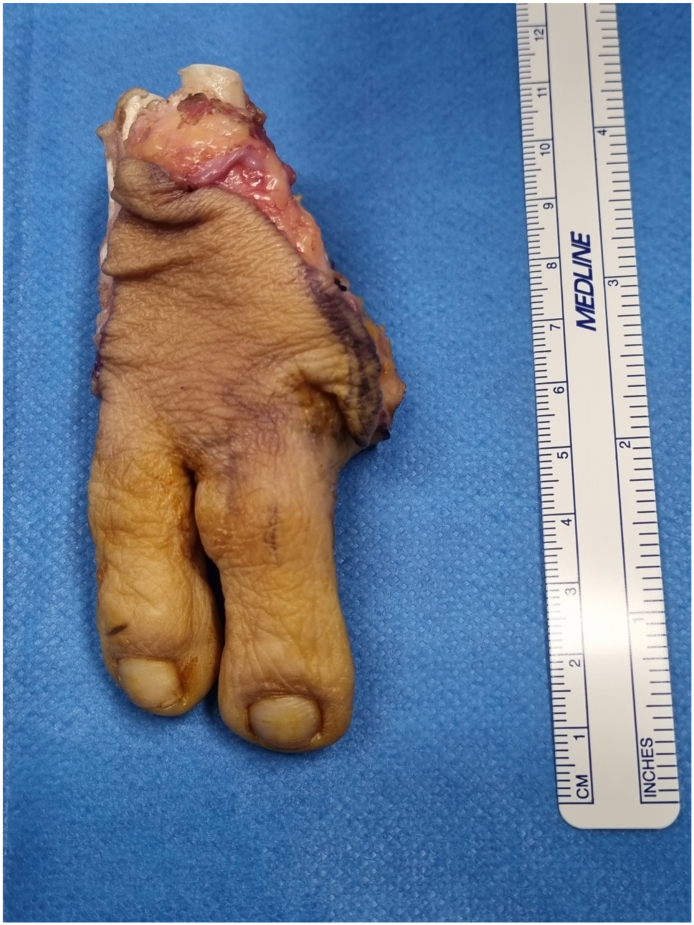
Intra operative piece.

**Fig. 4:**
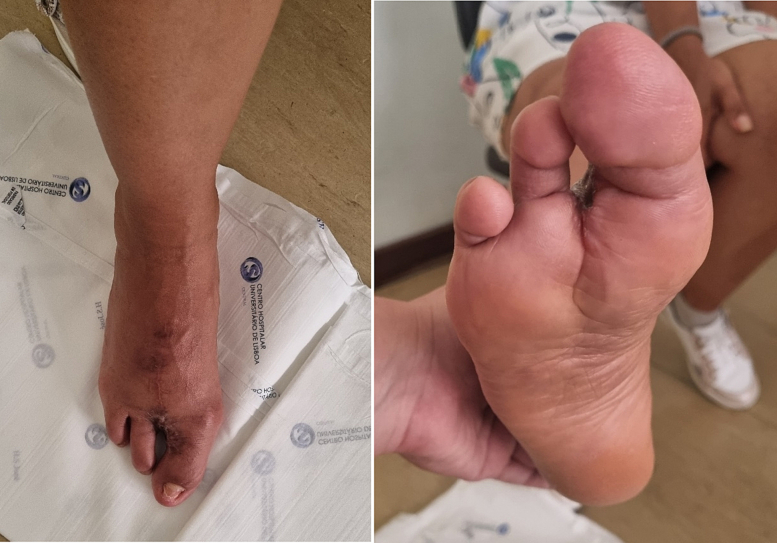
Post operative picture at 3 months.

Definitive pathological anatomy showed a complete excision of the tumor, with a 1,1 cm free margin. Immunohistochemical studies were outsourced and were not realized due to the lengthy decalcification process (Fig. 2, Fig. 3, Fig. 4).

**Figure:**
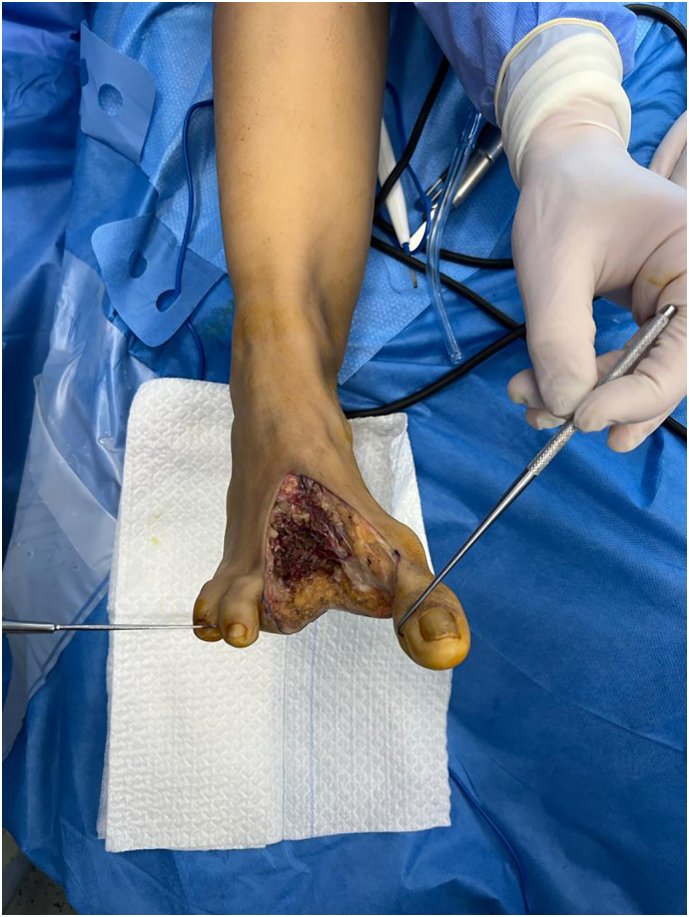

## 7 Discussion

Dermatofibrosarcoma protuberans (DFSP) is a rare and slow-growing type of skin cancer that arises from the dermal layer of the skin [1,2]. It typically presents as a painless, firm, and often irregularly shaped skin lesion that may grow slowly over many years [5]. While DFSP is generally considered a low-grade malignancy with a low risk of metastasis [2,5], it can be locally aggressive and difficult to treat if not diagnosed early and managed appropriately [19].

The main differential diagnosis is with dermatofibroma, atypical fibroxanthoma, cicatricial fibroma and Keloid scars, which are benign [3,9]. Rarer, neoplasic conditions that need to be excluded include fibrosarcoma, leiomyosarcoma, metastatic sarcoma, myxofibrosarcoma and angiosarcoma [9].

This clinical case showed an atypical location for the development of this disease. Through a combination of clinical examination, imaging studies, and histopathological analysis the diagnosis of DFSP was made. The primary treatment was surgical excision which required to finger ray amputation in order to ensure complete removal of the tumor and prevent local recurrence. In cases where surgical excision is not possible or would result in significant functional or cosmetic impairment, other treatment options such as radiation therapy or targeted therapy may be considered.

Recent advances in understanding the genetic and molecular basis of DFSP have led to the development of targeted therapies that specifically target the genetic mutations that drive tumor growth [2]. While these therapies are not curative and must be taken long-term, they have been shown to be effective in shrinking or stabilizing DFSP tumors in many patients.

## 8 Conclusion

While DFSP is a rare and often slow-growing skin cancer, it can be locally aggressive and difficult to treat if not managed appropriately. Early diagnosis and treatment with surgical excision with clear margins remain the gold standard of care, but newer treatment options such as radiation therapy and targeted therapy may be considered in cases where surgical excision is not possible or would result in significant functional or cosmetic impairment. Close monitoring and long-term follow-up are essential for all patients with DFSP to detect and manage any recurrence or metastasis.

## Abbreviations

DFSP: Dermatofibrosarcoma Protuberans

## Patient consent

Written informed consent was obtained from the patient for publication and any accompanying images. A copy of the written consent is available for review by the Editor-in-Chief of this journal on request.

## Funding

Nothing to declare.

## Ethical approva

Ethical exemption was given due to the single case study with patient data occultation and

written consent for publication.

## CRediT authorship contribution statement

**Miguel Matias:** Conceptualization, Methodology, Validation, Investigation, Data curation, Writing – original draft. **Miguel Verissimo:** Writing – review & editing, Visualization. **Raquel Barbosa:** Writing – review & editing, Visualization. **Diogo Casal:** Supervision.

## Declaration of competing interest

Nothing to declare.
